# Supplementary material for: Modulation of the Erwinia ligand-gated ion channel (ELIC) and the 5-HT3 receptor via a common vestibule site
Source: eLife. 2020 Jan 28;9:e51511. doi: 10.7554/eLife.51511 (PMC7015668; doi:10.7554/eLife.51511)
Supplement: Supplementary file 1. [file elife-51511-supp1.docx]

**Supplementary file 1. Crystallographic and refinement statistics.**

|  |  |  |
| --- | --- | --- |
|  | ELIC+PAM-Nb | ELIC+NAM-Nb |
| Crystallographic statistics |  |  |
| Beamline | PROXIMA 1 (SOLEIL) | X06A (SLS) |
| Wavelength (Å) | 0.97857 | 0.9999 |
| Spacegroup | *P*2_1_ | *P*1 |
| *a,b,c* (Å) | 105.6, 146.56, 140.24 | 121.73, 122.29, 128.15 |
| *α, β,γ* (°) | 90, 111.39, 90 | 71.02, 64.20, 61.66 |
| Resolution limits (Å) | 49.67 - 2.59 (2.65 - 2.59) | 48.32 - 3.25 (3.34 - 3.25) |
| *R_merge_* (%) | 6.1 (122.1) | 5.2 (95.4) |
| *R_meas_ (%)* | 7.8 (157.1) | 7.4 (134.9) |
| *R_pim_ (%)* | 4.9 (97.8) | 5.2 (95.3) |
| *<I/σ>* | 13.4 (1.0) | 5.5 (0.5) |
| *CC_1/2_ (%)* | 99.9 (45.8) | 99.7 (42.9) |
| Multiplicity | 4.8 (4.6) | 1.8 (1.6) |
| Completeness (%) | 99.7 (95.2) | 95.1 (89.4) |
| Total number of reflections | 594559 (26957) | 147646 (6664) |
| Number unique reflections | 123770 (5799) | 83183 (4160) |
|  |  |  |
| Refinement and model statistics |  |  |
| R_work_ (%) | 22.97 | 24.80 |
| R_free_ (%) | 24.59 | 26.43 |
| Rmsd bond distance (Å) | 0.008 | 0.008 |
| Rmsd bond angle (°) | 0.94 | 0.97 |
|  |  |  |
| Ramachandran analysis |  |  |
| Outliers (%) | 0.05 | 0.16 |
| Favored (%) | 97.16 | 96.53 |
|  |  |  |
| Poor rotamers (%) | 0.63 | 0.77 |
| Molprobity score | 1.54 (100rd percentile) | 1.78 (100rd percentile) |
|  |  |  |
|  |  |  |
