# Supplementary material for: Modulation of the Erwinia ligand-gated ion channel (ELIC) and the 5-HT3 receptor via a common vestibule site
Source: eLife. 2020 Jan 28;9:e51511. doi: 10.7554/eLife.51511 (PMC7015668; doi:10.7554/eLife.51511)
Supplement: Supplementary file 2. [file elife-51511-supp2.docx]

**Supplementary file 2: Structures used for structure-based superposition and Ω-loop analysis**

| Sequence identifier | PDB code | Reference DOI | Resolution (Å) | ECD or full-length |
| --- | --- | --- | --- | --- |
| **Ω-in conformation** |  |  |  |  |
| nAChα2 | 5fjv | [10.1073/pnas.1602619113](file:///Users/u0018409/Documents/Manuscripts/Erno21-22/revision-eLife/10.1073/pnas.1602619113) | 3.2 | ECD |
| nAChα3 | 6pv7, subunit A | [10.1016/j.neuron.2019.07.030](file:///Users/u0018409/Documents/Manuscripts/Erno21-22/revision-eLife/10.1016/j.neuron.2019.07.030) | 3.3 | FL |
| nAChα4 | 5kxi, subunit A | [10.1038/nature19785](file:///Users/u0018409/Documents/Manuscripts/Erno21-22/revision-eLife/10.1038/nature19785) | 3.9 | FL |
| nAChβ2 | 5kxi, subunit B | [10.1038/nature19785](file:///Users/u0018409/Documents/Manuscripts/Erno21-22/revision-eLife/10.1038/nature19785) | 3.9 | FL |
| nAChβ4 | 6pv7, subunit B | [10.1016/j.neuron.2019.07.030](file:///Users/u0018409/Documents/Manuscripts/Erno21-22/revision-eLife/10.1016/j.neuron.2019.07.030) | 3.3 | FL |
|  |  |  |  |  |
| **Ω-out conformation** |  |  |  |  |
| GABAβ1 | 6dw1, subunit B | [10.7554/eLife.39383](file:///Users/u0018409/Documents/Manuscripts/Erno21-22/revision-eLife/10.7554/eLife.39383) | 3.1 | FL |
| GABAβ2 | 6d6u, subunit B | [10.1038/s41586-018-0255-3](file:///Users/u0018409/Documents/Manuscripts/Erno21-22/revision-eLife/10.1038/s41586-018-0255-3) | 3.9 | FL |
| GABAβ3 | 6huj, subunit B | [10.1038/s41586-018-0832-5](file:///Users/u0018409/Documents/Manuscripts/Erno21-22/revision-eLife/10.1038/s41586-018-0832-5) | 3.0 | FL |
| GABAγ2L | 6huj, subunit C | [10.1038/s41586-018-0832-5](file:///Users/u0018409/Documents/Manuscripts/Erno21-22/revision-eLife/10.1038/s41586-018-0832-5) | 3.0 | FL |
| GABAα1 | 6huj, subunit A | [10.1038/s41586-018-0832-5](file:///Users/u0018409/Documents/Manuscripts/Erno21-22/revision-eLife/10.1038/s41586-018-0832-5) | 3.0 | FL |
| Glyα1 | 3jaf | [10.1038/nature14853](file:///Users/u0018409/Documents/Manuscripts/Erno21-22/revision-eLife/10.1038/nature14853) | 3.8 | FL |
| Glyα3 | 5tin | [10.1038/nsmb.3329](file:///Users/u0018409/Documents/Manuscripts/Erno21-22/revision-eLife/10.1038/nsmb.3329) | 2.6 | FL |
| GluCl | 3rhw | [10.1038/nature10139](file:///Users/u0018409/Documents/Manuscripts/Erno21-22/revision-eLife/10.1038/nature10139) | 3.2 | FL |
|  |  |  |  |  |
| **Ω-open conformation** |  |  |  |  |
| 5-HT3A | 6np0 | [10.1038/s41467-019-11142-8](file:///Users/u0018409/Documents/Manuscripts/Erno21-22/revision-eLife/10.1038/s41467-019-11142-8) | 2.9 | FL |
| Tmγ | 2bg9, subunit E | [10.1016/j.jmb.2004.12.031](file:///Users/u0018409/Documents/Manuscripts/Erno21-22/revision-eLife/10.1016/j.jmb.2004.12.031) | 4 | FL |
| Tmδ | 2bg9, subunit C | [10.1016/j.jmb.2004.12.031](file:///Users/u0018409/Documents/Manuscripts/Erno21-22/revision-eLife/10.1016/j.jmb.2004.12.031) | 4 | FL |
| Tmβ | 2bg9, subunit B | [10.1016/j.jmb.2004.12.031](file:///Users/u0018409/Documents/Manuscripts/Erno21-22/revision-eLife/10.1016/j.jmb.2004.12.031) | 4 | FL |
| Tmα | 2bg9, subunit A | [10.1016/j.jmb.2004.12.031](file:///Users/u0018409/Documents/Manuscripts/Erno21-22/revision-eLife/10.1016/j.jmb.2004.12.031) | 4 | FL |
| nAChα1 | 2qc1 | [10.1038/nn1942](file:///Users/u0018409/Documents/Manuscripts/Erno21-22/revision-eLife/10.1038/nn1942) | 1.9 | ECD |
| nAChα9 | 4d01 | [10.1038/nsmb.2900](file:///Users/u0018409/Documents/Manuscripts/Erno21-22/revision-eLife/10.1038/nsmb.2900) | 1.8 | ECD |
| α7-AChBP | 5ouh | [10.1074/jbc.M117.815316](file:///Users/u0018409/Documents/Manuscripts/Erno21-22/revision-eLife/10.1074/jbc.M117.815316) | 2.5 | ECD |
| ELIC | 6hjx | [10.1038/s41589-019-0369-4](file:///Users/u0018409/Documents/Manuscripts/Erno21-22/revision-eLife/10.1038/s41589-019-0369-4) | 2.5 | FL |
| GLIC | 4hfi | [10.1038/emboj.2013.17](file:///Users/u0018409/Documents/Manuscripts/Erno21-22/revision-eLife/10.1038/emboj.2013.17) | 2.4 | FL |
