# Supplementary material for: Modulation of the Erwinia ligand-gated ion channel (ELIC) and the 5-HT3 receptor via a common vestibule site
Source: eLife. 2020 Jan 28;9:e51511. doi: 10.7554/eLife.51511 (PMC7015668; doi:10.7554/eLife.51511)
Supplement: Supplementary file 3. [file elife-51511-supp3.docx]

**Supplementary file 3: Effects of MTSEA-biotin (MB) application on WT and mutant 5-HT_3_R.**

**A. MTS-sensitive mutants:**

| **Mutant** | ***p*EC_50_ (M)** | **5-HT EC_50_ (μM)** | **t-test p value**  **(- vs + MB *p*EC_50_)** | **n_H_** | **I_max_ /I_max_+MB** | **n** |
| --- | --- | --- | --- | --- | --- | --- |
| WT* | 5.73 ± 0.02 | 1.8 |  | 2.9 ± 0.3 |  | 9 |
| WT +MB* | 5.70 ± 0.05 | 2.0 | 0.585 | 3.5 ± 0.8 | 0.98 ± 0.03 | 3 |
| T112C | 5.17 ± 0.02 | 6.7 |  | 1.9 ± 0.2 |  | 4 |
| T112C +MB | 5.53 ± 0.02 | 2.9 | < 0.0001 | 2.5 ± 0.4 | 0.89 ± 0.03 | 4 |
| K149C | 5.21 ± 0.05 | 6.2 |  | 1.8 ± 0.3 |  | 7 |
| K149C +MB | 5.69 ± 0.09 | 2.1 | 0.0005 | 1.7 ± 0.7 | 0.96 ± 0.10 | 7 |
| L151C | 4.01 ± 0.03 | 98 |  | 1.7 ± 0.2 |  | 5 |
| L151C +MB | 4.96 ± 0.04 | 11 | < 0.0001 | 2.1 ± 0.5 | 11.1 ± 1.1^S^ | 5 |
|  |  |  |  |  |  |  |

Data = mean ± SEM; = * Data from Thompson et al. 2011 (1); ^S^ An ANOVA followed by a Dunnett’s multiple comparison test revealed that L151C was significantly different from WT (p < .0001).

**B. MTS-insensitive mutants:**

|  | WT* | Y86C | P111C | F125C | N147C |
| --- | --- | --- | --- | --- | --- |
| **I/I+MB** | 0.93 ± 0.06 | 0.96 ± 0.02 | 0.76 ± 0.07 | 0.77 ± 0.09 | 1.07 ± 0.12 |
| **n** | 3 | 4 | 4 | 3 | 3 |

Data = mean ± SEM; Values obtained using a single concentration of 5-HT (see methods for details); * Data from Thompson et al. 2011; An ANOVA followed by a Dunnett’s multiple comparison test revealed that no values were significantly different from WT (p = 0.054).
